# Effect of seasonal malaria chemoprevention on the acquisition of antibodies to *Plasmodium falciparum* antigens in Ouelessebougou, Mali

**DOI:** 10.1186/s12936-017-1935-4

**Published:** 2017-07-18

**Authors:** Almahamoudou Mahamar, Djibrilla Issiaka, Amadou Barry, Oumar Attaher, Adama B. Dembele, Tiangoua Traore, Adama Sissoko, Sekouba Keita, Bacary Soumana Diarra, David L. Narum, Patrick E. Duffy, Alassane Dicko, Michal Fried

**Affiliations:** 1 Malaria Research & Training Center, Faculty of Medicine, Pharmacy and Dentistry, University of Sciences Techniques and Technologies of Bamako, Bamako, Mali; 2 Laboratory of Malaria Immunology and Vaccinology, NIAID, NIH, 12735 Twinbrook Pkway Building TW3 Room 3W15, Rockville, MD 20852 USA

**Keywords:** Seasonal malaria chemoprevention, Seropositivity, Antibody

## Abstract

**Background:**

Seasonal malaria chemoprevention (SMC) is a new strategy to reduce malaria burden in young children in Sahelian countries. It consists of the administration of full treatment courses of sulfadoxine–pyrimethamine plus amodiaquine to children at monthly intervals during the malaria season. However, it is not clear if there is a cumulative effect of SMC over time on acquisition of antibodies to malaria antigens.

**Methods:**

A cross-sectional serosurvey was carried out 1 month after the last dose of SMC in 2016. Children aged 3–4 years were randomly selected from areas where SMC was given for 1, 2 or 3 years during the malaria season. Children in the areas where SMC had been implemented for 1 year but who failed to receive SMC were used as comparison group. Antibody extracted from dry blood spots was used to measure IgG levels to CSP, MSP-1_42_ and AMA1.

**Results:**

The prevalence of antibodies to AMA-1 were high and similar in children who received SMC for 1, 2 or 3 years and also when compared to those who never received SMC (96.3 vs 97.5%, adjusted OR = 0.99, 95% CI 0.33–2.97, p = 0.99). The prevalence of antibodies to MSP-1_42_ and to CSP were similar in children that received SMC for 1, 2 or 3 years, but were lower in these children compared to those who did not receive SMC (87.1 vs 91.2%, adjusted OR = 0.55, 95% CI 0.29–1.01, p = 0.05 for MSP-1_42_; 79.8 vs 89.2%, adjusted OR = 0.52, 95% CI 0.30–0.90, p = 0.019 for CSP).

**Conclusions:**

SMC reduced seropositivity to MSP-1_42_ and CSP, but the duration of SMC did not further reduce seropositivity. Exposure to SMC did not reduce the seropositivity to AMA1.

## Background

Malaria remains a major cause of morbidity and mortality, causing an estimated 235,000–639,000 deaths globally in 2015. Sub-Saharan Africa is disproportionately affected, suffering 92% of global malaria deaths with 88% occurring in children under 5 years of age [1], who are, therefore, the main target population for malaria control.

More than 200 million people live in areas of highly seasonal malaria transmission, where seasonal malaria chemoprevention (SMC) was recently recommended to prevent malaria disease and death in children. SMC consists of full treatment courses of sulfadoxine–pyrimethamine plus amodiaquine (SP + AQ) given to three-59 months old children at monthly intervals during the malaria season to maintain therapeutic anti-malarial drug concentrations in the blood throughout the period of greatest malaria risk [2]. Despite the substantial benefits provided by SMC, one concern is that SMC will impair the acquisition of protective immune responses, thereby increasing the risk of disease in later years. In Mali, children who received SMC over a single season experienced a small increase in clinical malaria during the following malaria transmission season compared to a control group [3, 4]. In Gambia, stopping chemoprophylaxis after a period of several years increased the risk of clinical malaria but did not result in a rebound in mortality in children [5]. In Mozambique, chemoprophylaxis with SP administered at 3, 4 and 9 months of age did not significantly modify antibody levels to *Plasmodium falciparum* erythrocytic-stage antigens in the first 2 years of life [6]. In Ghana, antibodies against various *P. falciparum* antigens were significantly lower in children treated once with SP than in untreated controls [7]. In Senegal, seropositivity rates to blood-stage antigens AMA-1 and GLURP were similar between children living in villages that implemented SMC compared to children that did not receive SMC, but antibody levels to both antigens were significantly higher in children that did not receive SMC [8].

The study hypothesis was that SMC would reduce immunity to blood-stage antigens (reflecting lower exposure from blood-stage infection) but not to liver-stage malaria antigens (reflecting exposure to infected mosquito bites). In this study, seropositivity rates and antibody levels to liver stage (CSP) and blood stage (MSP1, AMA1) antigens were measured and related these to the duration of SMC use.

## Methods

### Study site and procedure

The study was conducted in the health district of Ouelessebougou located 80 km south of Bamako, Mali. The target population was children aged three-59 months of age. SMC was implemented progressively in the district of Ouelessebougou. Eight sub-districts were randomly selected among the 13 sub-districts of Ouelessebougou to receive SMC over a period of 3 years: four sub-districts in 2014 (year 1); two sub-districts in 2015 (year 2); and two sub-districts in 2016 (year 3). During the third year of the study, the National Malaria Control Programme extended SMC to all parts of Mali, including the entire district of Ouelessebougou. Children in the selected areas received three rounds of SMC in the first year, and four rounds of SMC in the second and third years. During each round, children aged three-11 months received 75 mg of AQ given once daily for 3 days plus a single dose of 250/12.5 mg of SP, while children aged 12–59 months received 150 mg AQ base given once daily for 3 days and a single dose of 500/25 mg of SP. The single dose of SP was given only on the first day, at the same time as the first dose of AQ. Coverage of SMC by round was between 70 and 76% in study villages in 2014 and 84 and 90% in 2015 and 60 and 75% in 2016.

To assess the effect of SMC on the acquisition of the antibodies to falciparum malaria antigens, a cross-sectional survey have been conducted in December 2016 among children aged 3–5 years, randomly selected in areas where SMC was given for 1, 2 or 3 years. In each of these areas, six villages were randomly selected and their census lists were used for the selection of study children. Prior to this survey, children in the selected villages where SMC was implemented only for 1 year were surveyed to determine if they had received SMC or not; those who had not received any SMC were used as the comparison group.

After obtaining informed consent, children donated finger prick blood samples 1 month after last SMC round, for blood smear, haemoglobin concentration [haemoglobin analyzer HemoCue^®^ (Angelholm, Sweden)], and filter papers (Whatman^®^ protein saver cards, Z761575 ALDRICH) for extracting IgG. Dried filter papers were stored at −20 until use.

### Laboratories analysis

#### Antibody determination by ELISA

Two filter paper discs of 2.5 mm in diameter were taken from the centre of a single dried bloodspot and added into a deep well plate, incubated in 1120 µL of a 0.5% saponin solution at room temperature (RT). Plates were sealed and placed onto a plate shaker overnight. All samples were assayed for Immunoglobulin G to the recombinant proteins MSP-1_42_ (FVO strain) expressed in *Escherichia coli* [9], AMA-1 (3D7 strain) [10] and CSP-M3 expressed in *Pichia pastoris* [11] y. High-binding 96-well Immulon HBX4 microplates (Dynex Technologies, Inc) were coated with 200 ng per well of antigen diluted in 0.05 M carbonate-bicarbonate buffer and incubated overnight at 4 °C.

Plates were blocked with 5% skim milk in PBS for 1 h 30 min at room temperature. Plates were washed with 0.05% Tween 20 in PBS (PBS-Tween), samples and controls were added in duplicate, then incubated for 90 min at RT. Plates were washed with 0.05% Tween 20 in PBS and anti-Human IgG-HRP Conjugate (Promega, product number W4038) was added and plates were incubated for 1 h. After the plates were washed with 0.05% Tween in PBS, FAST OPD (Sigma, Product Number P9187) diluted in purified water was added, and the OD was measured at 450 nm.

#### Malaria parasitaemia

Thick blood smears were stained with 10% Giemsa for 15 min and read by certified microscopists. Asexual parasite densities were counted against 200 white blood cells (WBCs) assuming 8000 WBC/µL. A blood smear was considered to have negative results if no parasites were identified in 100 high-power fields. Slides were read by an experienced microscopist blinded to the treatment allocation. 10% of slides were re-read by a blinded expert reader for quality control.

### Statistical analysis

Data were entered and verified using DataFax. ELISA data were directly exported for analysis in StatView Version 5.0.1.0 (SAS Institute Inc) and Stata (version 12). Antibody seropositivity was considered the primary endpoint for this study, and antibody levels were treated as secondary endpoints. Proportions were compared using Chi square test. Associations between antibody seropositivity and SMC were assessed using logistic regression models. OD levels were compared between groups using Kruskal–Wallis test or Mann–Whitney U test in univariate analysis and linear regression models (after log transformation) were used to adjust for potential confounding factors (age, gender and malaria infection). The relationship between SMC and malaria infection was also assessed using logistic regression models. p-values less than or equal to 0.05 were considered significant.

## Results

### Study population characteristics

The analysis included 892 children aged 34–59 months. Some 158 participants never received SMC; 271, 232 and 231 participants received SMC respectively for 1, 2 and 3 years (Table 1). There were no differences in age, gender or haemoglobin concentration between groups. Prevalence of malaria infection was similar between children who received SMC for 1, 2 or 3 years (47.2, 43.1 and 37.2%, respectively p = 0.08) but was significantly higher in children that never received SMC (70.2%, p < 0.0001).

**Table 1:** Baseline characteristics

	No SMC (n = 158)	SMC 1Y (n = 271)	SMC 2Y (n = 232)	SMC 3Y (n = 231)	p
Age (months)					
Median (min–max)	50 (34–59)	47 (34–59)	47 (34–59)	47 (34–59)	0.44
Gender					
Male (%)	51.9	53.5	58.2	52.4	0.53
Parasite prevalence (%)	70.2	47.2	43.1	37.3	<0.0001

*No SMC* never received SMC, *SMC 1Y* received SMC 1 year, *SMC 2Y* received SMC 2 years, *SMC 3Y* received SMC 3 years, *n* number of subjects

### Anti-AMA-1 antibodies

**Fig. 1:**
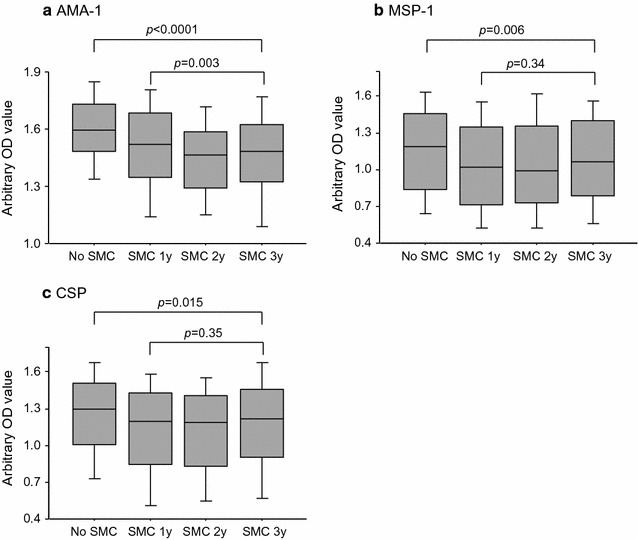
Comparison of antibody levels between children who never received SMC and children that received SMC. IgG levels to 3 malarial antigens in children who received SMC for 1–3 years and children who did not receive SMC were compared. The number of children in each group are as follow: No SMC, n = 158; SMC 1 year, n = 271; SMC 2 years, n = 232; SMC 3 years, n = 231

Anti-AMA-1 seroprevalence (Table 2) did not significantly differ between children that did not received SMC vs those that received SMC for 1 to 3 years (96.3 vs 97.5%, adjusted OR = 0.99, 95% CI 0.33–2.97, p = 0.99), nor did it differ between children who have received SMC for 1, 2 or 3 years (p = 0.16). In multivariate analysis, seropositivity was not related to the number of years of SMC (OR = 1.18, p = 0.79; OR = 1.45, p = 0.58; OR = 0.66, p = 0.49 for SMC for 1, 2 and 3 years, respectively, vs no SMC) after adjusting for age, gender and malaria infection (Table 2). However, median IgG levels were significantly lower in children who received any SMC vs those who never received SMC (p < 0.0001), and differed between children who have received SMC for 1, 2 and 3 years (p = 0.003) (Fig. 1). The differences in antibody levels between children who received any SMC and children that never received SMC remained significant after adjusting for age, gender and malaria infection (p = 0.005).

**Table 2:** Prevalence of AMA1 antibodies seropositivity in children who never received SMC and children that SMC for 1, 2 and 3 years

	n	%	95% CI	Unadjusted			Adjusted^a^		
				OR	95% CI	p	OR	95% CI	p
No SMC	154	97.5	95.0–99.9	Ref	**–**	**–**	Ref	**–**	**–**
SMC 1Y	263	97.0	95.1–99.1	0.85	0.25–2.88	0.79	1.18	0.34–4.07	0.79
SMC 2Y	226	97.4	95.4–99.5	0.97	0.27–3.52	0.97	1.45	0.39–5.37	0.58
SMC 3Y	218	94.4	91.4–97.4	0.43	0.13–1.36	0.15	0.66	0.21–2.15	0.49
SMC 1–3Y	707	96.3	94.9–97.6	0.68	0.23–1.97	0.47	0.99	0.33–2.97	0.99

*SMC 1Y* received SMC for 1 year, *SMC 2Y* received SMC for 2 years, *SMC 3Y* received SMC for 3 years, *SMC 1–3Y* received SMC for 1, 2, or 3 years

^a^Adjusted for age, gender and malaria infection

### Anti-MSP1 antibodies

Anti-MSP-1_42_ seroprevalence did not differ significantly between children who had received SMC for 1, 2 or 3 years (p = 0.36), but was significantly lower in children who received any SMC vs those who never received SMC (p = 0.02) (Table 3). After adjusting for age, gender and malaria infection, seroprevalence remained lower in children who received any SMC vs those who did never received SMC (OR = 0.55, 95% CI 0.29–1.01, p = 0.05) (Table 3).

**Table 3:** Prevalence of MSP1 antibodies seropositivity in children who never received SMC and children that SMC for 1, 2 and 3 years

	n	%	95% CI	Unadjusted			Adjusted^a^		
				OR	95% CI	p	OR	95% CI	p
No SMC	145	91.7	87.4–96.1	Ref	**–**	**–**	Ref	**–**	**–**
SMC 1Y	225	83.0	78.5–87.5	0.43	0.24–0.84	0.013	0.47	0.24–0.92	0.028
SMC 2Y	195	84.1	79.3–88.8	0.47	0.24–0.92	0.028	0.52	0.26–1.03	0.068
SMC 3Y	202	87.4	83.1–91.8	0.62	0.31–1.24	0.18	0.71	0.35–1.43	0.34
SMC 1–3Y	622	84.7	0.82–0.87	0.49	0.27–0.90	0.023	0.55	0.29–1.01	0.05

*SMC 1Y* received SMC for 1 year, *SMC 2Y* received SMC for 2 years, *SMC 3Y* received SMC for 3 years, *SMC 1–3Y* received SMC for 1, 2, or 3 years

^a^Adjusted for age, gender and malaria infection

Anti-MSP-1_42_ IgG levels did not significantly differ between children that received SMC for one, two or three years (p = 0.34), and were significantly lower in children who received any SMC vs those who never received SMC (p = 0.001) (Fig. 1). The difference remained significant in children who received SMC for 1, 2 or 3 years compared to children who did not receive SMC, after adjusting for age, gender and malaria infection (p = 0.005).

### Anti-CSP antibodies

Anti-CSP seroprevalence (Table 4) did not significantly differ between children who had received SMC for 1, 2 or 3 years (p = 0.06), but was significantly lower in children who received any SMC vs those who never received SMC (p = 0.007). After adjusting for age, gender and malaria infection, seroprevalence remained significantly lower in children who received SMC for 1 year (OR = 0.49, p = 0.02), 2 years (OR = 0.53, p = 0.04), or 3 years (OR = 0.54, p = 0.05) or in those who received SMC for any period of time (OR = 0.52, 95% CI 0.30–0.90, p = 0.019) (Table 4). IgG levels did not differ between children that received SMC for one, two or three years (p = 0.35), but were significantly lower in children who received any SMC vs those who never received SMC (p = 0.004) (Fig. 1). The difference in antibody levels remained significant after adjusting for age, gender and malaria infection in children who received SMC for one or two years (p = 0.025).

**Table 4:** Prevalence of CSP antibodies seropositivity in children who never received SMC and children that SMC for one, two and three years

	n	%	95% CI	Unadjusted			Adjusted^a^		
				OR	95% CI	p	OR	95% CI	p
No SMC	141	89.2	84.4–94.1	Ref	**–**	**–**	Ref	**–**	**–**
SMC 1Y	215	79.3	74.5–84.2	0.46	0.25–0.82	0.010	0.49	0.27–0.89	0.021
SMC 2Y	186	80.2	75.0–85.3	0.48	0.26–0.88	0.018	0.53	0.28–0.97	0.041
SMC 3Y	185	80.1	74.9–85.3	0.48	0.26–0.88	0.018	0.54	0.29–1.00	0.05
SMC 1–3Y	586	79.8	0.76–0.82	0.47	0.27–0.81	0.007	0.52	0.30–0.90	0.019

*SMC 1Y* received SMC for 1 year, *SMC 2Y* received SMC for 2 years, *SMC 3Y* received SMC for 3 years, *SMC 1–3Y* received SMC for 1, 2, or 3 years

^a^Adjusted for age, gender and parasitemia

### Malaria infection and IgG levels

All median IgG titres (anti-AMA-1, anti-MSP-1_42_ and anti-CSP) were significantly higher in malaria-infected than uninfected children (1.54 vs 1.47, p = 0.008 for AMA1; 1.09 vs 1.02 for MSP-1_42_, p = 0.03; 1.27 vs 1.15 for CSP, p = 0.0001), as were seroprevalences of IgG to all three antigens (Table 5). In logistic regression analysis, seroprevalence was significantly associated with positive blood smear, after adjusting for SMC status, age and gender (AMA-1: OR = 3.85, 95% CI 1.52–9.69, p = 0.004; MSP-1: OR = 1.54 95% CI 1.03–2.30, p = 0.03; CSP: OR = 1.54, 95% CI 1.08–2.19, p = 0.01).

**Table 5:** Malaria infection and seropositivity

	BS negative (%)	BS positive (%)	p value	Unadjusted			Adjusted^a^		
				OR	95% CI	p value	OR	95% CI	p value
AMA-1 seropositive	94.6	98.5	0.001	3.94	1.64–9.72	0.003	3.85	1.52–9.69	0.004
MSP-1 seropositive	83.3	88.9	0.015	1.61	1.09–2.37	0.016	1.54	1.03–2.30	0.03
CSP seropositive	77.9	85.4	0.0004	1.65	1.17–2.34	0.004	1.54	1.08–2.19	0.01

^a^Adjusted for age, gender and SMC as binary variable

## Discussion

This study evaluated the cumulative effect of SMC on acquisition of IgG to malarial antigens in children that received SMC for a varying number of years. IgG levels to two blood-stage antigens and the pre-erythrocytic antigen CSP were significantly higher in children who never received SMC vs those who did. Among children who received SMC, antibody levels to MSP-1_42_ and CSP were similar regardless of the number of years a child was exposed to SMC, but AMA-1 antibody levels were significantly higher in children that received SMC for one *versus* two and three years. Seroprevalence followed a similar pattern for IgG to CSP and MSP-1 but not IgG to AMA-1.

In this cohort of children, seroprevalences of IgG to all three malarial proteins were high, with the highest rates against AMA-1. AMA-1 is immunogenic and a limited exposure is sufficient to achieve antibody saturation against this antigen [12]. In this population, who were exposed to malaria for a limited number of years, malaria infections at the beginning and the end of the transmission season and possibly between treatment doses, are sufficient for seroconversion. Similarly in Senegal, seroprevalence of antibodies to AMA-1 and GLURP did not differ between children under the age of ten years who received SMC or not, although antibody levels were significantly higher in children that did not receive SMC [8].

Because the effect of SMC has been thought to be primarily directed against blood-stage parasites, the initially hypothesis was that SMC will not reduce antibody levels to sporozoite and liver-stage antigens, but instead found reduced IgG reactivity to the pre-erythrocytic antigen CSP. Pyrimethamine prophylaxis inhibits liver-stage development in a rodent model of malaria infection when given prior to sporozoite inoculation [13]. Because pyrimethamine inhibits the development of liver-stage falciparum parasites, this may result in a shorter exposure to CSP and other liver-stage antigens in children receiving SMC, thereby resulting in lower antibody levels to liver-stage antigens.

The strengths of our study include the following: children in the different groups are from the same area; selection for the phased implementation of SMC was done randomly and samples were collected at the same time and analysed together. Children who received SMC for one year and those who did not receive SMC were from the same villages, and the latter children reflect the coverage and compliance of the intervention in practice. We cannot rule out additional factors like socio-economic differences between children that did not receive SMC and those who received the intervention. A randomized controlled design with control villages where SMC was not implemented in 2016 could have potentially reduced such a selection bias, but would have been unethical as SMC was being implemented nation-wide in Mali in 2016.

## Conclusions

In this area of high seasonal malaria transmission, seroprevalence rates were less sensitive than IgG levels to detect differences between children that did or did not receive SMC. Exposure to SMC reduced antibody levels to AMA1, MSP-1_42_ and CSP. However, the duration of exposure to SMC had no effect on antibody levels to MSP-1_42_ and CSP.

## Abbreviations

AMA-1: apical membrane antigen 1
AQ: amodiaquine
CSP: circumsporozoite protein
ELISA: enzyme-linked immunosorbent assay
FVO: *Plasmodium falciparum* Vietnam Oak-Knoll
GLURP: glutamate-rich protein
CI: confidence intervals
IgG: immunoglobulin G
MRTC: Malaria Research and Training Center
SMC: seasonal malaria chemoprevention
MSP-1: merozoite surface protein 1
OD: optical density
OR: odd ratio
SP: sulfadoxine–pyrimethamine
USAID: US Agency for International Development
